# Optimization and Evaluation of Al^18^F Labeling Using a NOTA—or RESCA1-Conjugated AE105 Peptide Antagonist of uPAR

**DOI:** 10.3389/fnume.2021.799533

**Published:** 2021-12-13

**Authors:** Troels E. Jeppesen, Marina Simón, Josephine Torp, Line B. S. Knudsen, Julie Maja Leth, François Crestey, Michael Ploug, Jesper T. Jørgensen, Jacob Madsen, Matthias M. Herth, Andreas Kjaer

**Affiliations:** ^1^Department of Clinical Physiology, Nuclear Medicine & PET and Cluster for Molecular Imaging, Department of Biomedical Sciences, Rigshospitalet and University of Copenhagen, Copenhagen, Denmark; ^2^Department of Drug Design and Pharmacology, Faculty of Health and Medical Sciences, University of Copenhagen, Copenhagen, Denmark; ^3^Finsen Laboratory, Rigshospitalet, Copenhagen, Denmark; ^4^Biotech Research and Innovation Centre (BRIC), University of Copenhagen, Copenhagen, Denmark

**Keywords:** uPAR (urokinase plasminogen activator receptor), positron emision tomography (PET), Fluorine-18 (^18^F), imaging, peptide, RESCA1, NOTA, AE105

## Abstract

Fluorine-18 displays almost ideal decay properties for positron emission tomography (PET) and allows for large scale production. As such, simplified methods to radiolabel peptides with fluorine-18 are highly warranted. Chelation of aluminium fluoride-18 toward specific peptides represents one method to achieve this. With the current methods, chelation of aluminium fluoride-18 can be achieved using NOTA-conjugated peptides. However, the heating to 90–100◦C that is required for this chelation approach may be deleterious to the targeting moiety of the probe. Recently, a new chelator, RESCA1, was developed allowing Al^18^F chelation at room temperature. Here, we optimize the labeling procedure enabling high chelation efficacy of fluoride-18 at 22◦C, even at full batch labeling. The optimized procedure was tested by Al^18^F-labeling of RESCA1-AE105—a uPAR targeting peptide. NOTA-AE105 was also labeled with Al^18^F, and the two peptides were compared head-to-head. [^18^F]AlF-NOTA-AE105 and [^18^F]AlF-RESCA1-AE105 could be produced in equal radiochemical yields (RCY), radiochemical purities (RCP) and molar activities. Additionally, the two peptides showed comparable binding affinity to uPAR and uptake in cells expressing the uPAR, when evaluated *in vitro*. Overall, we found that the performances of [^18^F]AlF-NOTA-AE105 and [^18^F]AlF-RESCA1-AE105 were grossly comparable, but importantly RESCA1 can be labeled with aluminium fluoride-18 at 22◦C. Consequently, this study showed that RESCA1 is superior to NOTA with respect to Al^18^F chelation of temperature sensitive molecules, such as thermolabile peptides and proteins as well as that full batch chelation of RESCA1 with fluoride-18 is possible.

## Introduction

For PET imaging, peptides, and other smaller biomolecules are often labeled with ^68^Ga, exemplified by [^68^Ga]Ga-DOTA-TOC/TATE (1), due to the ease of radiolabelling and access to ^68^Ga/^68^Ge generators. The generator is, however, not suited for large scale production and therefore it would be advantageous if ^18^F-labeling of the targeting probe was feasible. Labeling with ^18^F normally requires elevated temperature, non-aqueous solvents and basic conditions, not suited for most biomolecules (2). A direct labeling approach with Al^18^F was previously demonstrated, where aluminium is coordinated in a chelator (typically NOTA or similar TACN-based chelators) and bound to ^18^F (3). This labeling approach proceeds in aqueous media at pH 4–5 with heating to 90–110°C (4). The need for heating was, importantly, unnecessary using the chelator RESCA1 (5), but rigorous optimization has to be performed when labeling at lower concentrations of RESCA1, and to enable full-batch labeling, using all radioactivity from one fluorine-18 production. Furthermore, the pharmacokinetics of RESCA1 coupled to a peptide have not been tested and compared to NOTA *in vitro*. Biodistribution in healthy animals with an [^18^F]AlF-RECA1-affibody, an [^18^F]AlF-RESCA1-nanobody and [^18^F]AlF-RESCA1-HSA have been studied previously (5). In the present study, the peptide AE105, which is an antagonist for uPA-binding to its receptor uPAR, was used as the targeting moeity (6). High uPAR-expression in lesion sites and shed to plasma correlates with poor prognosis and metastatic disease in several types of cancer (7). Labeling with Al^18^F of RESCA1 was optimized and compared to NOTA, and Al^18^F labeling of RESCA1-AE105 and NOTA-AE105 was performed, compared and evaluated in a cell-binding assay.

## Materials and Methods

Chemicals were purchased from Sigma-Aldrich unless stated otherwise. NCS-MP-NODA (NOTA) were purchased from Chematech mdt., Dijon, France. Analytical HPLC was performed on a Thermo Fischer Ultimate 3000 system and a gamma detector (Scansys Laboratorieteknik) connected in series. Data collection and liquid chromatography control used the program Chromeleon 7.2. Analytical HPLC was performed using a gradient from 0 to 50% B over 5 min., 50 to 100% B over 1 min., 100% B over 1 min., 100 to 30% B over 1 min., A: H_2_O 0.1% TFA, B: MeCN 0.1% TFA, on a Phenomenex Luna C18(2) column (5 μm, 150 × 4.6 mm), flow 1 mL/min. NMR was performed on a 600 MHz Bruker Avance III HD, or a 400 MHz Bruker Avance III. All data are presented as ± SD where applicable.

### Synthesis

#### (1*R*,2*R*)-N1-Benzylcyclohexane-1,2-Diamine (1)

A solution of benzyl bromide (1.09 g, 6.3 mmol) in MeCN (40 mL) was added dropwise to a solution of trans-1,1-diaminocyclohexane (7.13 g, 62.4 mmol) in MeCN (10 mL), and the solution was stirred overnight at room temperature. The substance was concentrated *in vacuo*, dissolved in CH_2_Cl_2_ (10 mL) and washed 8 times with sat. Na_2_CO_3(aq.)_ (12 mL) and H_2_O (12 mL). The organic phase was dried over MgSO_4_ and concentrated *in vacuo* to afford **1** as a light-yellow oil (0.90 g, 70%). ^1^H-NMR was in accordance with previously published data (8). ^1^H-NMR (400 MHz, CDCl_3_) 7.32–7.15 (m, 6H), 3.89 (d, J = 13.1 Hz, 1H), 3.64 (d, J = 13.0 Hz, 1H), 2.33 (ddd, J = 11.0, 9.1, 4.1 Hz, 1H), 2.13–1.97 (m, 2H), 1.89 – 1.77 (m, 1H), 1.75 – 1.59 (m, 1H), 1.53 (s, 4H), 1.21 (dtt, J = 22.1, 12.8, 3.2 Hz, 2H), 1.14–0.86 (m, 2H).

#### di-*Tert*-Butyl 2,2′-(((1*R*,2*R*)-2-(Benzyl(2-(*Tert*-Butoxy)-2-Oxoethyl)Amino)Cyclohexyl)Azanediyl) Diacetate (2)

DIPEA (3.9 mL, 22.4 mmol) was added to a solution of **1** (1.41 g, 6.9 mmol) in CH_2_Cl_2_ (15 mL), and stirred for 15 min. To the resulting mixture, *tert*-butyl bromoacetate (3.3 ml, 22.5 mmol) was added slowly and stirred for 16 h at room temperature. The substance was concentrated *in vacuo*, and the crude product was dissolved in CH_2_Cl_2_ (10 mL) and washed three times in sat. NaHCO_3__(aq.)_ (12 mL) and H_2_O (12 mL). The organic phase was dried over MgSO_4_, and purified by silica dry column chromatography using a gradient of 19:1–2:1 Heptane:EtAOAc to afford **2** as a light yellow oil (2.33 g, 61%). ^1^H-NMR was in accordance with previously published data (9). ^1^H-NMR (400 MHz, CDCl_3_) 7.48–7.38 (m, 2H), 7.32–7.28 (m, 2H), 7.25–7.17 (m, 1H), 3.97 (dd, J = 24.9, 13.4 Hz, 1H), 3.76–3.65 (m, 1H), 3.52–3.22 (m, 5H), 2.75–2.67 (m, 1H), 2.54 (td, J = 10.7, 3.5 Hz, 1H), 2.10–1.98 (m, 2H), 1.76–1.61 (m, 3H), 1.52–1.37 (m, 27H), 1.32–0.85 (m, 6H).

#### RESCA1

Synthesis of RESCA1 from **2** was realized as described earlier (9). Briefly, the deprotection was performed in TFA, yielding RESCA1. ^1^H-NMR was in accordance with the previously published data (9).

#### di-*Tert*-Butyl 2,2′-(((1*R*,2*R*)-2-((2-(*Tert*-Butoxy)-2-Oxoethyl)Amino)Cyclohexyl)Azanediyl)Diacetate (3)

**2** (2.33 g, 4.23 mmol) was dissolved in MeOH (20 mL) and degassed with N_2_ for 5 min. 10% Pd/C (0.46 g, 0.43 mmol) was added to the solution and stirred overnight under H_2_ (1 atm.). The suspension was filtered through Celite and the substance was concentrated *in vacuo* affording **3** (1.54 g, 79%). ^1^H-NMR was in accordance with previously published data (8). ^1^H-NMR (400 MHz, CDCl_3_) 3.52–3.20 (m, 7H), 2.35 (dtd, J = 23.8, 10.0, 3.6 Hz, 2H), 2.05–1.86 (m, 2H), 1.81–1.57 (m, 1H), 1.45 (d, J = 1.5 Hz, 27H), 1.31–0.94 (m, 4H).

#### 4-((((1*R*,2*R*)-2-(Bis(2-(*Tert*-Butoxy)-2-Oxoethyl)Amino)Cyclohexyl)(2-(*Tert*-Butoxy)-2-Oxoethyl)Amino)Methyl)Benzoic Acid (4)

To a solution of **3** (1.54 g, 3.7 mmol) and DIPEA (1.2 mL, 6.9 mmol) in CH_2_Cl_2_ (12 mL), bromomethyl benzoic acid (0.73 g, 3.4 mmol) was added dropwise. The resulting solution was stirred overnight at room temperature. The substance was concentrated *in vacuo* and the crude product was purified by preparative HPLC [C18 (250 × 21 mm)] using a gradient of 30–100% MeCN/H_2_O in 25 min, with a flow of 20 mL/min. The collected fractions were lyophilized to afford **4** (0.90 g, 45%). ^1^H-NMR was in accordance with previously published data (8). ^1^H-NMR (400 MHz, CDCl_3_) 8.13–7.87 (m, 2H), 7.56 (dd, J = 12.0, 7.6 Hz, 2H), 4.12 (s, 1H), 3.75 (s, 1H), 3.53–3.22 (m, 6H), 2.61 (d, J = 66.2 Hz, 2H), 2.11–1.91 (m, 4H), 1.67 (s, 2H), 1.41 (t, J = 10.3 Hz, 27H), 1.26–0.92 (m, 6H).

#### RESCA1-AE105 and NOTA-AE105

The conjugation of AE105 with t-butoxy RESCA1 (**4**) was performed by solid phase peptide synthesis at ABX (Radeburg, Germany) as a contract synthesis. NOTA-AE105 was purchased from ABX (Radeburg, Germany) as a custom synthesis. AE105 (Asp-Cha-Phe-(D)Ser-(D)Arg-Tyr-Leu-Trp-Ser) with conjugation at the N-terminal.

### Surface Plasmon Resonance

#### Kinetics

Binding kinetics of the peptides-uPAR interactions were determined with surface plasmon resonance (SPR) on a Biacore T200™ system (Cytiva), as outlined (10). In brief; 10 μg/mL uPAR^1−283^ in 10 mM sodium acetate pH 5.0 was covalently immobilized on a CM5 sensor chip via amine coupling yielding a surface density of 905-1,333 RU (~26–39 fmol/mm^2^) (11). Subsequently, we measured the binding kinetics of the various peptides to immobilized uPAR with single cycle protocols in which the peptides were injected as five serial 2-fold dilutions for 200 s with a short dissociation phase in between (100 s). The last analyte injection was followed by a 1,000 s long dissociation phase. In the end of each cycle, two consecutive injections of 0.1 M acetic acid in 0.5 M NaCl regenerated the sensor chip. All experiments were run with a flowrate of 50 μl/min in 10 mM HEPES, 150 mM NaCl, 3 mM EDTA, and 0.05% (v/v) surfactant P-20 at pH 7.4 at 20°C. The kinetic rate constants (*kon* and *koff*) as well as the KD (*koff/kon*) were determined by non-linear regression fitting of the curves to a simple bimolecular interaction model. We applied the BiacoreT200 Evaluation^TM^ 3.0 software for the global fitting. Additional binding kinetic curves is provided in the Supplementary Material.

#### IC50

The IC_50_-values of RESCA-AE105, NOTA-AE105, AE105 (Asp-Cha-Phe-(D)Ser-(D)Arg-Tyr-Leu-Trp-Ser) and AE105-mut (Asp-Cha-Glu-(D)Ser-(D)Arg-Tyr-Leu-Glu-Ser) on the uPAR·uPA interaction were determined with SPR on a Biacore 3000™ instrument or a Biacore T200™ system (Cytiva). The set-up is overall as described previously (12). In brief, we obtained a high surface density of pro-uPA^S356A^ by immobilizing >5,000 RU (~ 0.1 pmol pro-uPA/mm^2^) on a CM5 sensor chip. This results in a heavily mass transport limited reaction, which causes the observed association rates (ν_obs_) to be directly proportional to the concentrations of binding active uPAR in solution (when low concentrations of uPAR are tested—here 0.06 nM to 2 nM). For the analysis, we incubated 2 nM uPAR with a 3-fold dilution series of the peptides (ranging from 0.076 nM to 1.5 μM) and the ν_obs_ was measured for 300 s at 20°C with a flow rate of 50 μL/min. The running buffer contained 10 mM HEPES, 150 mM NaCl, 3 mM EDTA and 0.05 % (v/v) surfactant P20, pH 7.4. The sensor chip was regenerated with two consecutive injections of 0.1 M acetic acid, 0.5 M NaCl in the end of each cycle. In parallel, we measured a standard curve (2-fold dilution of uPAR covering 0.06–2 nM). This standard curve included one repeated concentration point at the end to validate the biological integrity of the sensor chip.

### Radiochemistry

Chromafix PS-30 cartridges were used without pre-conditioning. All other cartridges were pre-conditioned with EtOH (5 mL) and H_2_O (10 mL) prior to use. iTLC-SG plates (Agilent) developed in MeCN/H_2_O 3:1 was used for radioTLC analysis. The RadioTLC were analyzed using a Cyclone Plus system (Perkin Elmer). Fluorine-18 was produced by an ^18^O(p,n)^18^F reaction on a MC32 Scanditronix or on a RDS Eclipse, CTI/Siemens apparatus. RCY's reported are decay corrected. Isolated activity yields are non-decay corrected. Apparent molar activities are calculated from the isolated activity and 3–5 consecutive HPLC analyses of NOTA-AE105 or RESCA1-AE105 with a known concentration and compared to the purified [^18^F]AlF-NOTA-AE105 or [^18^F]AlF-RESCA1-AE105.

#### Al^18^F Labeling of RESCA1 and NODA

^18^F-water was split in aliquots and applied to a QMA cartridge (Cl^−^ form) and eluted with 0.1 M NaOAc (500 μL). The eluate was further divided in aliquots, before AlCl_3_ (2 mM, 9–180 nmol, 0.1M NaOAc) was added, and left at room temperature for 10 min. RESCA1 (2 mM, 10–200 nmol, 0.1M NaOAc/EtOH 1:1) or NODA (2 mM, 10–200 nmol, 0.1M NaOAc/EtOH 1:1) and EtOH (to 50% of total volume) were added and the mixture was shaken at room temperature or 90°C for 15 min. Analysis was conducted with the analytical HPLC method setup and on radioTLC. An example of an HPLC analysis is provided in the Supplementary Material.

#### Elution Studies for Al^18^F Labeling of RESCA1

^18^F-water was split in aliquots and applied to a QMA cartridge (Cl^−^ form) or a Chromafix PS-30 (${\text{HCO}}_{3}^{-}$ form), and eluted with the specified elution solvent, either 0.9% NaCl (300 μL), 0.1M NaOAc (500 μL), 1M NaOAc (225 μL) or 20% NaCl (300 μL). The eluted solution was split in 3 and diluted with the elution solvent to 223.5 μL. AlCl_3_ (2 mM, 34 nmol, 17 μL, 0.1M NaOAc) was added, and left at room temperature for 10 min. RESCA1 (2 mM, 37.5 nmol, 18.8 μL, 0.1M NaOAc/EtOH 1:1) and EtOH (240.5 μL) were added, and the mixture was shaken at room temperature for 15 min. Analysis was conducted on the analytical HPLC setup and on radioTLC.

#### Al^18^F Labeling of RESCA1-AE105 and NOTA-AE105

For the handling of higher radioactive amounts, a suction setup was used, exemplified in the Supplementary Material. ^18^F-water was applied to a QMA cartridge (Cl^−^ form), and eluted with 0.9% NaCl (300 μL). AlCl_3_ (60 nmol, 30 μL, 2 mM in 0.1M NaOAc) was added and the reaction mixture was left at room temperature for 5 min. RESCA1-AE105 (120 nmol, 60 μL, 0.1M NaOAc/EtOH 1:1) or NOTA-AE105 (120 nmol, 60 μL, 0.1M NaOAc/EtOH 1:1) and EtOH (330 μL) was added, and the mixture was reacted at room temperature (for RESCA1-AE105) or at 90°C (for NOTA-AE105), for 12 min. The resulting reaction mixture was diluted with 10 mL H_2_O, prior to application to a tC2 light cartridge (Waters). The labeled peptide was eluted with EtOH (0.5 mL) and diluted with 9.5 mL PBS. The final product was analyzed with the analytical HPLC setup. An example of an HPLC analysis is provided in the Supplementary Material.

#### LogD Determination

LogD was determined by the shake-flask method, as previously described (13). Briefly, Octanol and water phases were saturated by mixing the two and shaking overnight. The phases were separated prior to use. Either [^18^F]AlF-NOTA-AE105 or [^18^F]AlF-RESCA1-AE105 was diluted 1:100 in PBS. The peptide was mixed with PBS to a total volume of 200 μL. Octanol (200 μL) was added, and the mixture was shaken for 30 min. The mixture was spun down in a low-speed centrifuge. Fifty microliters of each layer was aspired and counted in a gamma counter. LogD was calculated from the following formula, where CPS_o_ is the CPS from the octanol phase, and the CPS_w_ is the CPS from the water phase. Each determination was performed in triplicates.


$$\begin{array}{l} LogD_{7.4}=log\left(\frac{CPS_{o}}{CPS_{w}}\right) \end{array}$$

### *In vitro* Cell Uptake

The human glioblastoma cell line U-87 MG was cultured in Dulbecco's Modified Eagle's medium (DMEM) with 10% foetal bovine serum and 1% penicillin/streptomycin at 37°C and 5% CO_2_. The tongue squamous cell carcinoma cell line OSC-19.luc2 (and OSC-19.luc2 uPAR KO), kindly provided by prof. J.N. Myers, M.D. Anderson Cancer Centre, Texas, USA, were cultured in DMEM medium supplemented with 10% foetal bovine serum, 1% penicillin/streptomycin, 1% sodium pyruvate and 1% non-essential amino acids. At ~70% confluence, cells were harvested and transferred to a 96-well-plate (Nunclon Delta surface, Thermo Scientific) at a density of 30,000 cells/well. The cell binding assay was performed the following day. The cells were first washed with PBS and then incubated for 2 h at 4°C with 200 nM of either [^18^F]AlF-RESCA1-AE105 or [^18^F]AlF-NOTA-AE105 in incubation buffer (PBS with 1% bovine serum albumin). AE105 (1,000-fold) was also added to some of the wells right before the tracer in order to estimate non-specific binding. Afterwards, the cells were washed, harvested from the plate and the cell-bound radioactivity was measured in a Gamma Counter (Wizard2, Perkin Elmer).

## Results and Discussion

**Figure 1:**
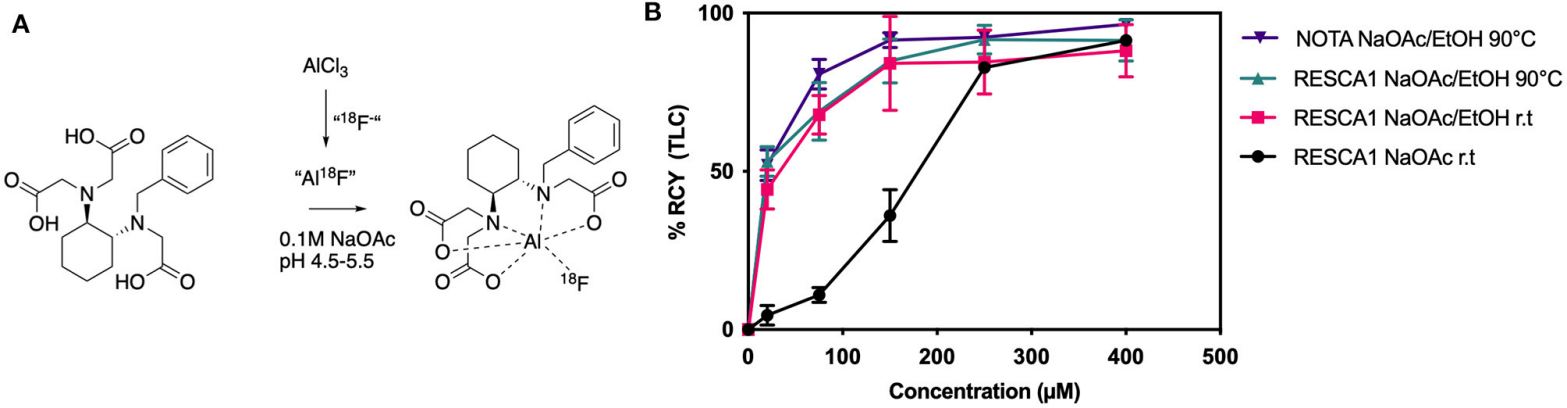
**(A)** Al^18^F chelation with RESCA1. Typical conditions listed (9). **(B)** Radiochemical yield of Al^18^F chelation by RESCA1 or NOTA, determined by TLC, at different concentrations and conditions. r.t.: room temperature (22◦C) (*n* = 3).

RESCA1 has previously been demonstrated to give a high radiochemical yield (RCY) when coupled to Al^18^F at a high concentration of RESCA1 (typically 150 μM, 1 mL), Figure 1A (9). However, optimization is needed to render labeling at lower concentrations applicable. To that end, RESCA1 was synthesized as described earlier (9) with minor modifications, see experimental section. The concentration dependence of Al^18^F labeling with RESCA1 at room temperature was compared to NOTA (containing only two acid pendant arms) at 90°C, Figure 1B. The radiochemical yield (RCY, determined by radioTLC), was comparable for NOTA and RESCA1, when 50% EtOH and 0.1M NaOAc was used as reaction media. A lower RCY for RESCA1 was obtained when 50% EtOH was omitted. Heating of the Al^18^F reaction with RESCA1 did not improve the RCY and is therefore not required. Notably, Al^18^F labeling of RESCA1 gives a comparable yield at all concentrations measured to Al^18^F labeling of NOTA, but importantly this was accomplished at a substantially lower reaction temperature.

**Figure 2:**
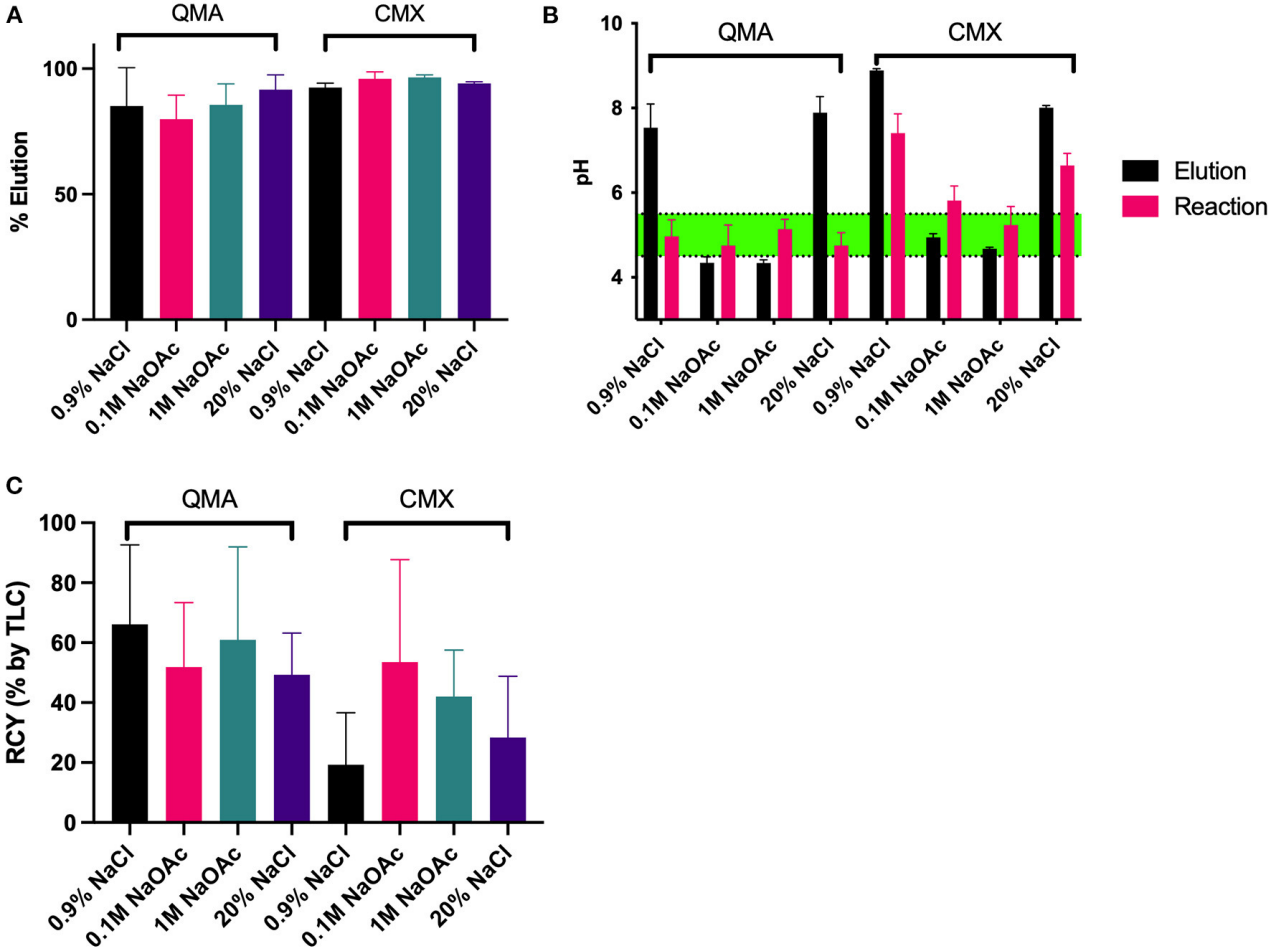
Elution, pH and radiochemical yield (RCY) parameters for RESCA1 with different elution solutions. **(A)** Elution percentage from either QMA (Cl^−^ form) or Chromafix PS-30 (CMX, ${\text{HCO}}_{3}^{-}$ form) with the specified solutions. **(B)** pH of the resulting elution, and pH of the reaction mixture for the specified elution solutions, where 50% EtOH, AlCl_3_, and RESCA1 were added, as described in the experimental section. **(C)** RCY (determined by radioTLC) of the reactions with the different elution solutions. Reactions were carried out with 75 μM RESCA1, and 50% EtOH for 15 min at 22◦C. See experimental section for details.

Optimization of cartridge type, elution solution and pH were performed for concentrating fluorine-18, Figure 2. Chromafix PS-30 cartridges (CMX, ${\text{HCO}}_{3}^{-}$ form) generally elute a slightly higher percentage of the applied activity, Figure 2A. However, the ${\text{HCO}}_{3}^{-}$ form makes it difficult to control the pH after elution and, therefore, in the resulting reaction mixture. The pH measured in 3 out of 4 reactions applying elution solutions for CMX cartridges were higher than the optimal pH for Al^18^F chelation in RESCA1 (9), while all applied solutions for QMA were within the optimal pH range, Figure 2B. In the end, that resulted in slightly higher RCY (determined by radioTLC) for Al^18^F chelation in RESCA1 for elutions from QMA, compared to CMX cartridges. However, a large spread of RCY is seen. 0.9% NaCl eluted from a QMA cartridge results in a solution where it is easy to control pH, gives a high RCY and was therefore used for further RESCA1-AE105 labeling.

**Figure 3:**
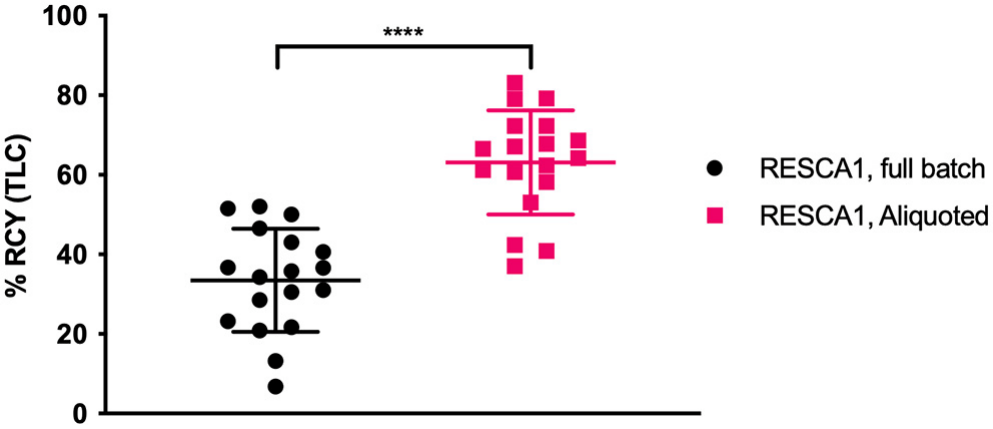
Radiochemical yield (RCY, determined by radioTLC) of full batch and aliquoted Al^18^F labeling of RESCA1, 75 μM, 50% EtOH/0.1M NaOAc. *n* = 36, ****p < 0.0001, unpaired *t*-test.

**Figure 4:**
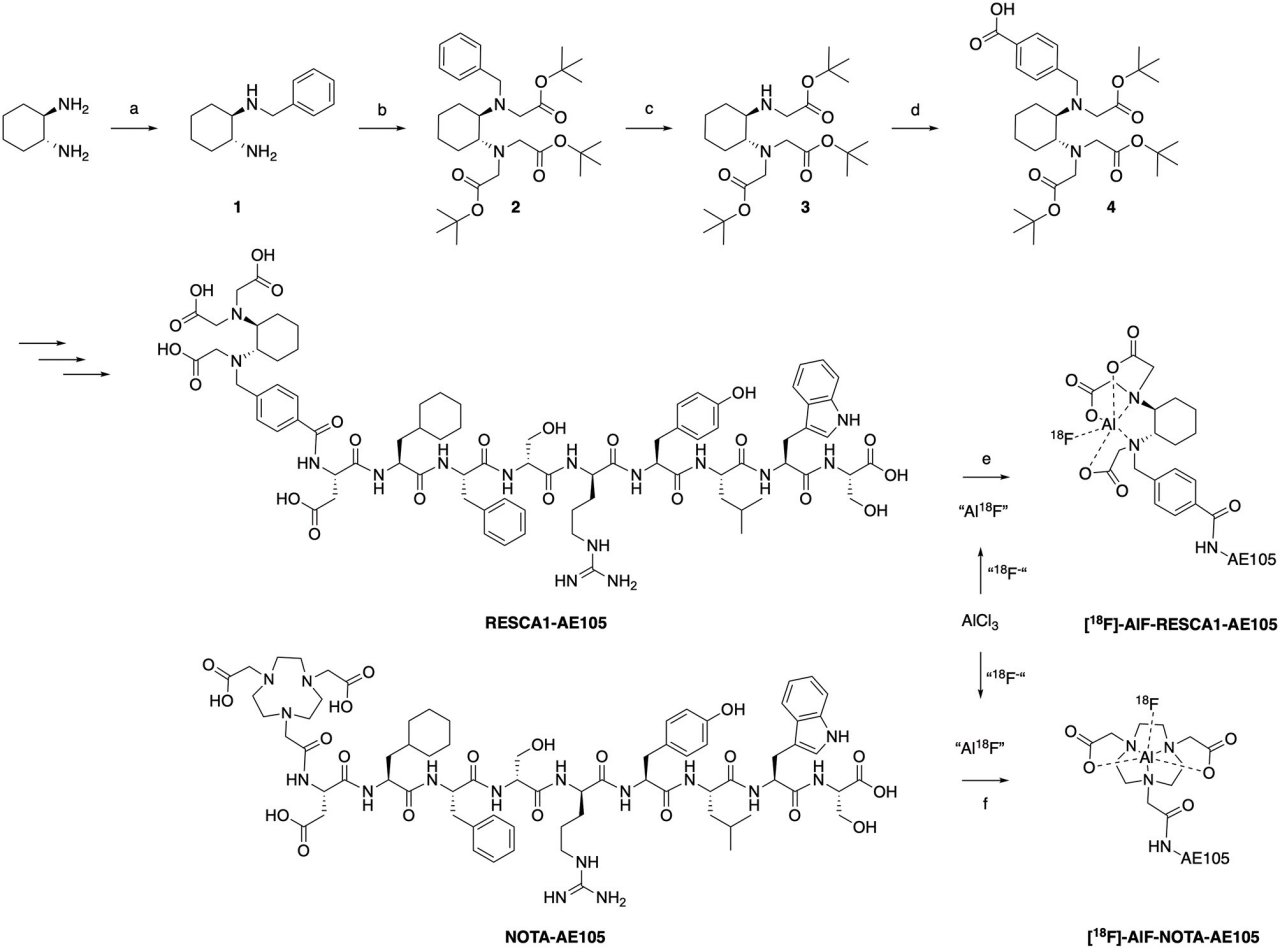
Synthesis of RESCA1-AE105. (a) Benzyl bromide, MeCN, r.t. (b) *tert*-butyl bromoacetate, DIPEA, CH_2_Cl_2_, r.t. (c) 10% Pd/C, MeOH, H2 (1 atm) (d) Bromomethyl benzoic acid, DIPEA, CH_2_Cl_2_, r.t. (e) 1. AlCl_3_, ^18^F^−^, NaOAc 0.1M, pH 4–4.5, 5 min r.t. 2. “Al^18^F,” 0.1M NaOAc, pH 4.5–5.5, EtOH (50% vol.), 12 min, r.t. (f) 1. AlCl_3_, ^18^F^−^, NaOAc 0.1M, pH 4–4.5, 5 min r.t. 2. “Al^18^F,” 0.1M NaOAc, pH 4.5–5.0, EtOH (50% vol.), 12 min, 90◦C.

RESCA1 was labeled with Al^18^F either with an aliquot labeling or with a full batch labeling. For aliquot labeling, aliquotation was performed after cartridge concentration. For full batch labeling, all the acquired target water was using in a single reaction. Otherwise, the reaction setup was identical. In general, the full batch labeling method gave lower radiochemical yields (analyzed by radioTLC) compared to the aliquoted labeling method, Figure 3. One possible explanation is that the ^18^F target water contains contaminants including boron, silicon, and aluminium, originating from the cyclotron, the tubings to the hotcell, from the cartridges or from the glass/plasticware used. To further verify that target water has an influence, direct labeling with ^18^F target water was performed (without an anion exchange step) and this does indeed lower the yields, as does labeling where decayed ^18^F target water is added to the labeling mixture (Supplementary Material). Most cationic metal contaminants from the cyclotron and tubings are eliminated in the anion exchange step, but boron, silicon and aluminium from cartridges or glassware can still be problematic (14). All the contaminants, whether originating from the target and tubing or from cartridges or glassware, will be diluted in an aliquoted setup, explaining the difference in RCY. Since a solution to the problem is not the aim of this article, the issue was not investigated further.

t-Butoxy RESCA1 (**4**) was produced as described earlier (9) with small modifications, Figure 4. *Trans*-1,1-diaminocyclohexane was mono-benzylated to afford the diamine (**1**). The t-butoxy compound (**2**) was synthesized from diamine (**1**) by a nucleophilic substitution reaction with *tert*-butyl bromoacetate. The benzyl-group was replaced by reducing the amine over Pd/C to afford compound (**3**), followed by the addition of benzoic acid to yield t-butoxy RESCA1 (**4**). The conjugation of AE105 with t-butoxy RESCA1 (**4**) was performed by solid phase peptide synthesis at ABX (Radeburg, Germany) as a contract synthesis.

**Figure 5:**
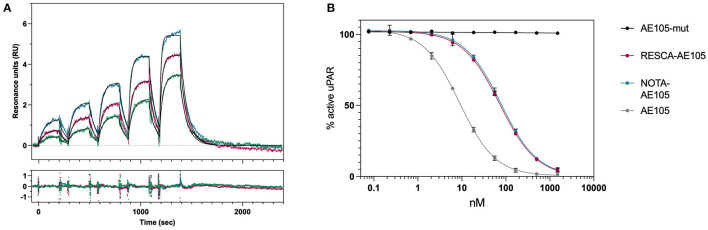
Binding kinetics and IC_50_ determination for AE105 compounds. **(A)** Sensorgrams obtained from five serial 2-fold dilution injections of RESCA-AE105. Different colors of the sensorgrams represent three different serial injections of RESCA-AE105: 3.13–50 nM (*green*), 6.25–100 nM (*pink*), and 12.5–200 nM (*teal*). The black lines represent the global fits to a simple biomolecular interaction model. Residual plot is shown below the sensorgram. **(B)** Competition of the uPA-uPAR interaction by different AE105-peptides. Additional sensorgrams and fits are in the Supplementary Material and details in the experimental section.

To determine if the conjugations to NOTA or RESCA1 have any influence on the interaction with uPAR, we used surface plasmon resonance (SPR) to determine the binding properties between RESCA1-AE105 and uPAR. This was accomplished by measuring both its direct real-time binding kinetics to immobilized uPAR and its competitive inhibition on uPAR-binding to its biological ligand, uPA, immobilized at a high density on a CM5 sensor chip, Table 1, Figure 5. The results showed that RESCA1-AE105 and NOTA-AE105 exhibited comparable binding kinetics to uPAR with equal association (*k*_*on*_) and dissociation (*k*_*off*_) rate constants, and with equal K_D_ and IC_50_. Thereby RESCA1—and NOTA-AE105 are comparable in binding affinity to uPAR. AE105mut is used as negative control, and AE105 is used as positive control. The conjugation of the large chelators, NOTA or RESCA1, to the small uPAR-targeting 9-mer AE105 has as expected, a penalty on the binding to uPAR, but the interactions are still in the low nM range (15, 16).

**Table 1:** Binding data from SPR of, NOTA-AE105, RESCA1-AE105, AE105 (positive control), and AE105mut (negative control).

**Substance**	**k_on_**	**k_off_**	**K_D_**	**IC_50_**
	**(× 10^5^ M^−1^s^−1^)**	**(x 10^−3^ s^−1^)**	**(nM)**	**(nM)**
AE105	10.10 ± 0.03	4.13 ± 0.01	4.10	8.86 ± 0.30
AE105mut	NB	NB	NB	>> 10^3^
NOTA-AE105	2.24 ± 0.01	12.50 ± 0.02	55.7	72.4 ± 1.52
RESCA1-AE105	2.92 ± 0.01	14.00 ± 0.02	48.0	76.7 ± 3.23

*The association (k_on_) and dissociation (k_off_) rate constants and the equilibrium dissociation constant K_D_ for the interaction between the peptides RESCA-AE105, NOTA-AE105, AE105, and AE105-mut in solution and immobilized uPAR. IC50 values were obtained by fitting to a four-parameter dose-response model, n = 3. Standard errors (shown as ±) are derived from the global fitting procedure. NB = no binding. AE105-mut shows no measurable binding up to 200 nM for the kinetic measurements*.

[^18^F]AlF-RESCA1-AE105 and [^18^F]AlF-NOTA-AE105 were produced in high RCY, with an acceptable apparent molar activity, and a high purity, Table 2. LogD was measured for both compounds by the shake flask method. The LogD was lower for [^18^F]AlF-NOTA-AE105, but both peptides have a logD value between −2 and −2.4, making them very hydrophilic of nature, Table 2.

**Table 2:** Radiochemical analysis of Al^18^F labeling of RESCA1-AE105 and NOTA-AE105.

**Substance**	***n***	**Starting activity (GBq)**	**Yield (MBq)**	**RCY**	**Apparent molar activity (GBq/μmol)**	**Purity (HPLC)**	**LogD**
[^18^F]AlF-RESCA1-AE105	7	2.4–8.3	2,338 ± 965	55 ± 13%	33.1 ± 13.7	>99%	−2.05 ± 0.09
[^18^F]AlF-NOTA-AE105	4	1.9–8.8	1,909 ± 814	41 ± 2%	29.9 ± 12.3	>99%	−2.38 ± 0.03

*Yield is isolated yield. Radiochemical yield (RCY) is determined from starting activity (decay corrected) and isolated yield. LogD was determined with the shake flask method as described in the experimental section*.

**Figure 6:**
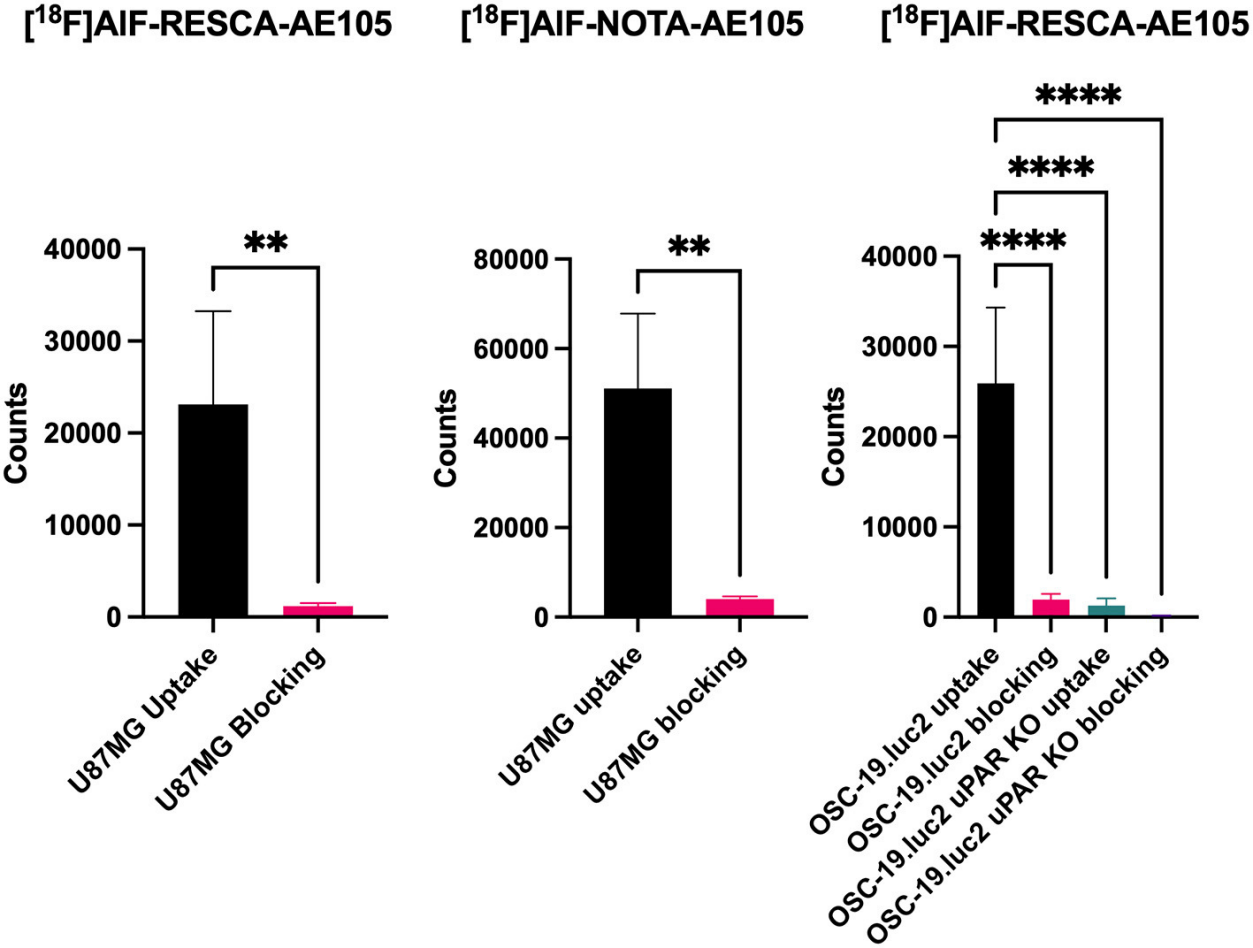
Cell uptake and blocking with AE105 (1,000-fold) in U-87 MG (high uPAR expression) for [^18^F]AlF-RESCA1-AE105 and [^18^F]AlF-NOTA-AE105. Cell uptake and blocking with AE105 (1,000-fold) in OSC-19.luc2 (high uPAR expression) and OSC-19.luc2 uPAR KO for [^18^F]AlF-RESCA1-AE105. *n* = 4/5 per group. Data shown as mean ± SD. **p < 0.01, unpaired *t*-test. ****p < 0.0001, one-way ANOVA.

The binding of [^18^F]AlF-RESCA1-AE105 and [^18^F]AlF-NOTA-AE105 to uPAR was confirmed by *in vitro* cell binding assays. Specific uptake and blocking (with AE105) were investigated in U-87 MG (high uPAR expressing) cells, Figure 6. We observed a significant blocking effect when incubating cells with AE105 prior to addition of radiolabelled peptide. Uptake and blocking were comparable for both peptides. In addition, uptake of the new peptide, [^18^F]AlF-RESCA1-AE105, was further investigated in OSC-19.luc2 (high uPAR expressing) cells, and OSC-19.luc2 cells with a uPAR-encoding gene knocked out, OSC-19.luc2 uPAR KO, Figure 6. Here, a clear uptake was seen in OSC-19.luc2 cells, which could be blocked almost completely with AE105 incubation. Further, uptake in OSC-19.luc2 uPAR KO cells was on level with blocked uptake in OSC-19.luc2 cells, thereby confirming the specific binding to uPAR.

The stability of the [^18^F]AlF-RESCA1-AE105 was not examined in this work. However, the stability *in vivo* of the NOTA-AE105 has been confirmed with several different isotopes (15, 17), and the stability of the [^18^F]AlF-RESCA1 motif itself has been shown in plasma (9), and *in vivo*. Therefore, the combined [^18^F]AlF-RESCA1-AE105 is suspected to be stable *in vivo* as well.

Together, these results indicate that the same radiochemical parameters (RCY, molar activity, RCP) can be achieved for Al^18^F labeling of RESCA1-AE105 and of NOTA-AE105. This is significant because not all peptides can withstand heating at 90–110°C required for Al^18^F labeling of a NOTA-based peptide. The two ^18^F labeled peptides behave very similar, making it possible to exchange a NOTA chelator for a RESCA1 chelator in heat-sensitive peptides for Al^18^F labeling by using the described procedure.

## Conclusion

In conclusion, we have optimized conditions for full batch labeling of RESCA1-peptides with Al^18^F and used these for labeling of [^18^F]AlF-RESCA1-AE105. This new peptide was compared to the known [^18^F]AlF-NOTA-AE105, and were found similar in terms of RCY, RCP, and molar activity. Furthermore, the two labeled peptides show similar uptake and blocking in U-87 MG (uPAR positive) cells. Uptake and blocking of [^18^F]AlF-RESCA1-AE105 in OSC-19.luc2 (uPAR positive) cells, and OSC-19.luc2 uPAR KO cells was performed to corroborate the specificity. Al^18^F chelation in the RESCA1 chelator can be performed at room temperature, which paves the way for use of RESCA1 in heat sensitive peptides and proteins. Further, the uptake and blocking in cells implies that [^18^F]AlF-RESCA1-AE105 is a specific uPAR tracer.

## Data Availability Statement

The original contributions presented in the study are included in the article/Supplementary Material, further inquiries can be directed to the corresponding author/s.

## Author Contributions

The organic synthesis was conducted by JT and FC, under supervision of MH. Radiochemical synthesis was performed by TJ and LK, under supervision of JM and AK. Cell studies was performed by MS under supervision of JJ. SPR measurements was conducted by JL under supervision of MP. The manuscript was written by TJ, MS, JT, and JL with input from all authors. The study was designed by all authors and have read and agreed to the published version of the manuscript.

## Funding

This project received funding from the European Union's Horizon 2020 research and innovation programme under grant agreements no. 670261 (ERC Advanced Grant) and 668532 (Click-It), the Lundbeck Foundation, the Novo Nordisk Foundation, the Innovation Fund Denmark, the Danish Cancer Society, Arvid Nilsson Foundation, Svend Andersen Foundation, the Neye Foundation, the Research Foundation of Rigshospitalet, the Danish National Research Foundation (grant 126), the Research Council of the Capital Region of Denmark, the Danish Health Authority, the John and Birthe Meyer Foundation and Research Council for Independent Research. AK is a Lundbeck Foundation Professor.

## Conflict of Interest

The authors declare that the research was conducted in the absence of any commercial or financial relationships that could be construed as a potential conflict of interest.

## Publisher's Note

All claims expressed in this article are solely those of the authors and do not necessarily represent those of their affiliated organizations, or those of the publisher, the editors and the reviewers. Any product that may be evaluated in this article, or claim that may be made by its manufacturer, is not guaranteed or endorsed by the publisher.
