# Supplementary material for: Optimization and Evaluation of Al18F Labeling Using a NOTA—or RESCA1-Conjugated AE105 Peptide Antagonist of uPAR
Source: Front Nucl Med. 2021 Dec 13;1:799533. doi: 10.3389/fnume.2021.799533 (PMC11440976; doi:10.3389/fnume.2021.799533)
Supplement: Supplementary file 1 [file Data_Sheet_1.docx]

Supplementary Material

# SPR kinetic data

Binding kinetics between AE105 (A), NOTA-AE105 (B) and AE105-mut (C) and immobilised uPAR as assessed by SPR, supplementary Figure 1.

**A**

**B**

**C**

**Supplementary Figure 1.** Sensorgrams obtained from five serial 2-fold dilution injections of the peptides. For AE105: Different colours of the sensorgrams represent two different serial injections of 0.8-12.5 nM (*blue*) and 1.6-25 nM (*purple*). For NOTA-AE105 and AE105-mut: Different colours of the sensorgrams represent three different serial injections of 3.13-50 nM (*green*), 6.25-100 nM (*purple*) and 12.5-200 nM (*teal*). The black lines represent the global fits.

# Suction setup for labeling of RESCA1-AE105 and NOTA-AE105

A simple manual suction setup was used for handling of higher amounts of radioactivity used for Al^18^F labeling of RESCA1-AE105 and NOTA-AE105. The suction setup is depicted and described below

Receiving vial, and reaction vial were acid washed, and cleaned with metal free water prior to use

AlCl_3_: 30 µL, 60 nmol, 83 µM

Chelator: 60 µL, 120 nmol, 167µM

EtOH: 330 µL (50%)

The dilution vial was filled with 10 mL H_2_O. The PBS vial is filled with 9.5 mL PBS. tC2light and QMA cartridges were preconditioned as described in the materials and methods section. All the tubings, vials and needles were setup prior to synthesis start and placed behind lead shielding in a hotcell. The NaCl vial was filled with a NaCl solution (300 µL, 0.9% in water). The reaction vial was filled with the AlCl_3_ solution. The EtOH vial was filled with 500 µL EtOH. The heating block were set to 90°C for NOTA conjugation. For RESCA1 conjugation, the heating block was left off. A long piece of tubing was connected to a 50 mL syringe that could be operated outside the hotcell to generate the negative pressure needed to transfer solvents and reaction mixtures.

**Receiving and transferring ^18^F to QMA**

^18^F-water was transferred to the receiving vial from the cyclotron. ^18^F was transferred to QMA by suction.

**Eluting ^18^F and forming Al^18^F**

The QMA with tubings and needles were transferred to the NaCL vial and the reaction vial. NaCl was used to elute 18F from the QMA into the reaction vial. The reaction vial was left at room temperature for 5 minutes.

**Addition of chelator and EtOH**

The chelator solution as mixed with EtOH (330 µL). The mixture was transferred to the reaction vial with a syringe and a coated metal needle. All tubings to the reaction vial was removed. For RESCA1 chelation, the reaction was left at room temperature for 12 minutes. For NOTA chelation, the reaction vial was heated at 90°C for 12 minutes.

**Dilution of reaction**

The reaction mixture was diluted, by transferring it to the dilution vial, with a ventilation needle in the reaction vial.

**Purify by tC2 light**

The tube connecting the dilution vial and the reaction vial was removed and the tube connecting to the seppak setup (shown above) was connected. The two 3-way valved was adjusted to connect the dilution vial, through the tC2 light cartridge, to the waste vial. The syringe used for suction was now used to exert positive pressure in the dilution vial to press the liquid to the waste vial. The 3-way valves were adjusted to connect the EtOH vial, via the tC2 light cartridge, the the PBS vial. Postive pressure was used to elute the final Al^18^F-labelled peptide into the PBS formulation.

# Target water validation

This experiment was set up to test the influence of metal contaminants from the cyclotron/tubings and the cartridges used.

This was achieved by performing an aliquoted Al18F chelation with RESCA1, diluted with decayed cyclotron water (received the previous day, and left for decay overnight), metal free water, or 0.1 M NaOAc (as used for the elutions). The RCY (by iTLC) was compared as output.

Decayed cyclotron water was received the previous day and allowed to decay overnight. One part was used for dilution directly. Another part was concentrated on QMA (preconditioned as described in the experimental section) and eluted with 0.1 M NaOAc (600 µL). A third part was concentrated on a Chromafix-PS cartridge (preconditioned as described in the experimental section) and eluted with 0.1 M NaOAc (600 µL).

Metal free water was prepared as described for the decayed cyclotron water to serve as a reference. 0.1 M NaOAc was used for reference elutions as well (as used in the experimental section).

The reaction with RESCA1 is essentially the same as described in the experimental section. ^18^F was concentrated on a chromafix PS-30 cartridge, washed with 5 mL metal free water, and eluted with 0.1 M NaOAc (600 µL). An aliquoted reaction was performed, by reacting 50 µL ^18^F, diluted with cyclotron water, metal free water or 0.1 M NaOAc (174 µL), with AlCl_3_ (33.75 nmol, 2 mM in 0.1 M NaOAc) for 10 min at room temperature. Then RESCA1 (37.5 nmol, 2 mM in 0.1 M NaOAc/EtOH 50%) and EtOH (241 µL, to a total of 50%) was added and the reaction mixture was shaken for 15 min. The RCY was recorded by iTLC, as described in the experimental section.

Each reaction was performed in at least triplicates and depicted below. A one-way ANOVA model was used for statistics.

**Supplementary Figure 2.** Dependence of RCY on metal impurities from cartridges or from the cyclotron and related tubings. *: p<0.05, **: p<0.01, ***: p<0.001, ****: p<0.0001. One-way ANOVA.

These results show a trend of lower RCY when metal free water is passed through a cartridge, confirming the notion that metal impurities exist in these cartridges. Further, decayed cyclotron water gives a significant lower yield compared to metal free water. This means that metal contaminants stem from the cyclotron and/or tubing to the outlet in the hotcell, and from cartridges used. In the most extreme cases, the RCY can be halved if cyclotron water is used directly, without removing some of the metal impurities by a cartridge.

This effect is probably also the reason for the lower yield of full batch labelling, compared to aliquoted labelling, as described in the article.

# Representative radio-HPLC chromatograms

Radio-HPLC chromatograms are depicted in supplementary figure 3 for [^18^F]AlF-RESCA1, [^18^F]AlF-NOTA, [^18^F]AlF-RESCA1-AE105 and [^18^F]AlF-NOTA-AE105. Details regarding the gradient and equipment used is described in the materials and methods section.

**Supplementary Figure 3.** Representative radio-HPLC chromatograms for a) [^18^F]AlF-RESCA1, b) [^18^F]AlF-NOTA, c) [^18^F]AlF-RESCA1-AE105 and d) [^18^F]AlF-NOTA-AE105. The red line represents the radio-trace and the black line represents the UV-trace, on the left and right y-axis respectively.
